# Pombiliti and Opfolda: shaping the future of adult late-onset pompe disease: an editorial

**DOI:** 10.1097/MS9.0000000000002483

**Published:** 2024-08-22

**Authors:** Rumaisa Riaz, Ajeet Singh, Laiba Shakeel, Laveeza Fatima, Aymar Akilimali

**Affiliations:** a Internal Medicine, Dow University of Health Sciences (DUHS), Karachi; b Department of Internal Medicine, Allama Iqbal Medical College, Lahore, Pakistan; c Department of research, medical research circle (MedReC), Goma, DR Congo

## Background

Pompe disease, also called glycogenosis type II or acid maltase deficiency, is an uncommon and persistent neuromuscular disorder characterized by the progressive weakening of skeletal and cardiac muscles^1^. While estimates of the disease’s incidence vary from 1:40 000 to 60 000 individuals, the reported incidence differs across populations^2^. This article focuses on POMBILITI (cipaglucosidase alfa) + OPFOLDA (miglustat), an FDA-approved dual-component therapy. It thoroughly examines the treatment’s pharmacokinetics, recommended dosage, mode of action, and the prospective advantages it may offer in addressing late-onset Pompe disease in adults. The objective is to provide valuable insights into this therapeutic approach, enhancing comprehension of its potential benefits for individuals grappling with the condition.

## Pathophysiology

**Figure 1:**
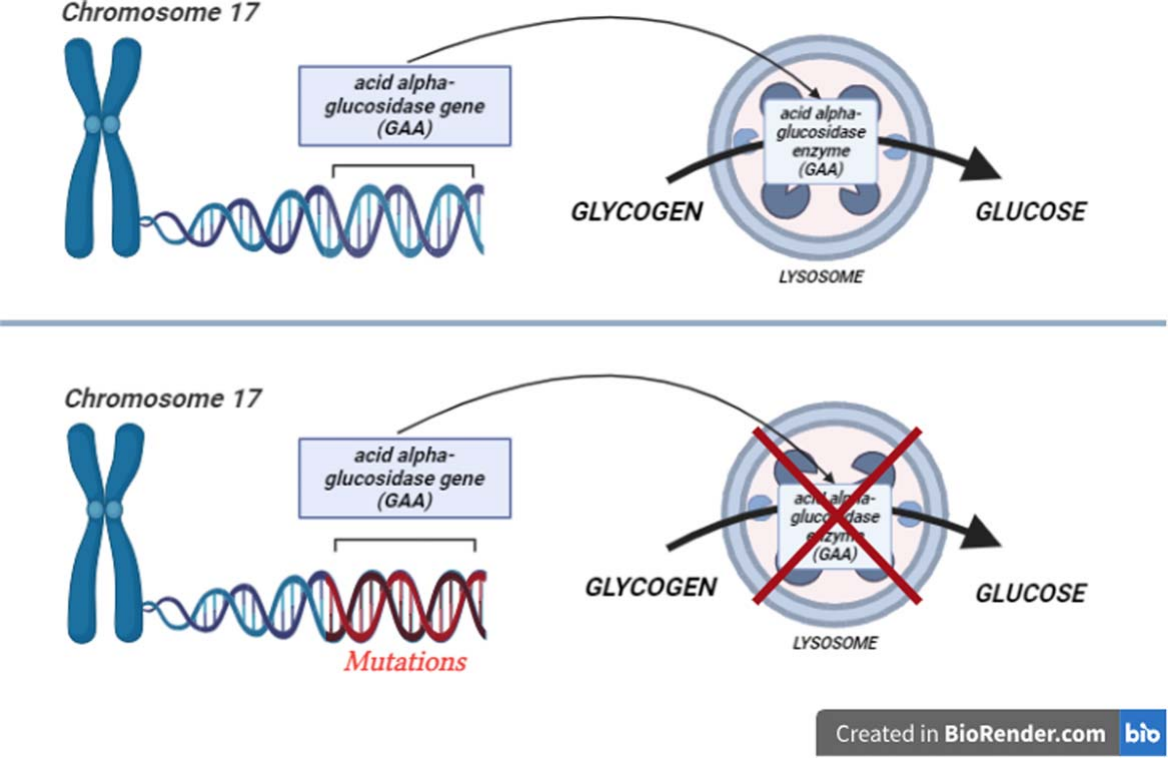
Pathophysiology of Pompe disease. (Created with biorender.com).

Pompe disease (PD) is an autosomal recessive condition arising from mutations in the acid alpha-glucosidase gene (GAA) located on chromosome 17, encoding the lysosomal GAA enzyme, which is responsible for the conversion of glycogen into glucose, a critical energy source for muscle function. This enzyme deficiency, whether partial or total, leads to an abnormal buildup of glycogen, serving as the underlying cause of Pompe’s disease^1^. This mechanism by which glycogen builds up is shown in Fig. 1. Glycogen is an intracellular polymer consisting of glucose residues joined in linear chains by α 1→4 bonds and branches joined at branch sites by α 1→6 bonds^3^. Glycogen accumulation occurs in lysosomes across various tissues, with skeletal and cardiac muscles primarily affected, resulting in clinical symptoms. The condition manifests with diverse signs, ranging from hypertrophic cardiomyopathy and hypotonia in infancy to a gradual skeletal muscle myopathy in adults. Muscle structure and strength decline due to progressive lysosomal enlargement, rupture, cytoplasmic glycogen accumulation, and myofibril displacement. Recent research underscores multiple pathogenic mechanisms, including autophagy, oxidative stress, mitochondrial abnormalities, and calcium homeostasis, contributing to tissue damage in Pompe disease and similar lysosomal storage disorders. Non-contractile substances, such as glycogen-filled lysosomes, cytoplasmic glycogen pools, autophagic remnants, and lipofuscin, disrupt the contractile machinery, ultimately causing muscle injury and reduced performance^1^. Although this condition manifests as a single disease continuum, two distinct phenotypes are widely accepted. The early onset, the infantile form, is marked by a profound or near complete deficiency of GAA. Symptoms emerge within the initial months of life, presenting as feeding difficulties, poor weight gain, dyspnea, muscle weakness, an enlarged heart, floppiness, and head lag. In the absence of prompt and appropriate treatment, many infants affected by this form do not survive beyond their first year, succumbing to cardiac or respiratory complications.

Conversely, the late onset, often called the juvenile or adult form, results from a partial deficiency of GAA. This form primarily affects skeletal muscles and progresses over several years, ultimately leading to death due to respiratory failure. In this phenotype, the heart is typically not affected^4^.

## Current enzyme replacement therapies for Pompe disease

### Alglucosidase alfa

**Figure 2:**
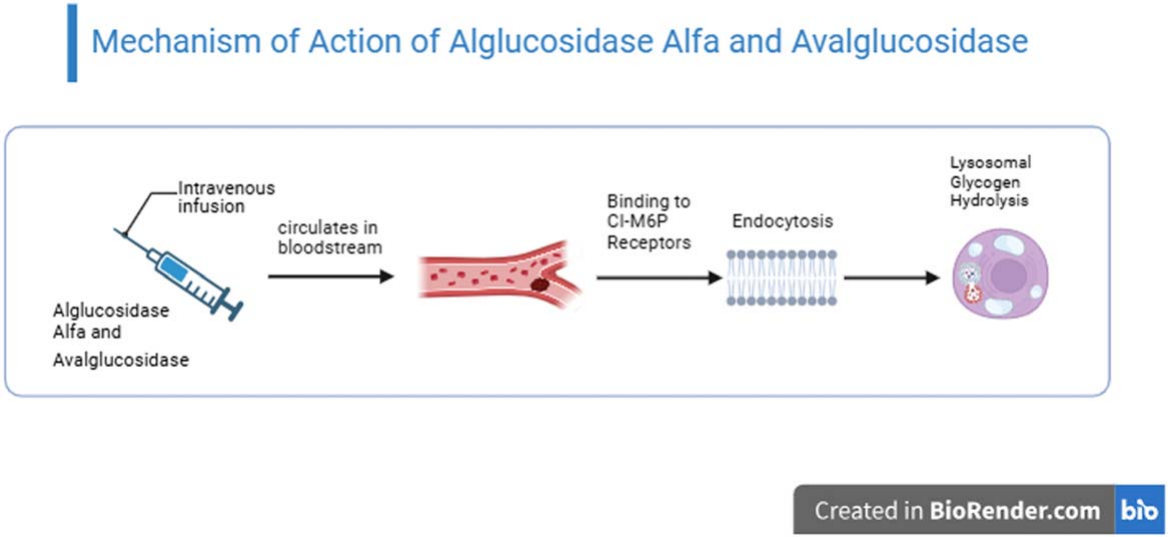
Mechanism of action of Alglucosidase alfa and Avalglucosidase. (Created with biorender.com).

In the absence of prompt treatment following diagnosis, the patient outcomes deteriorate with the likelihood of wheelchair use and respiratory support augmenting by 13% and 8% each year, respectively^5^. In 2006, alglucosidase alfa received breakthrough approval as the first enzyme replacement therapy based on the intravenous infusion of recombinant human GAA for treating IOPD patients^6^. Following promising outcomes in IOPD patients, a randomized, placebo-controlled trial (LOTS trial) was conducted to evaluate the efficacy of alglucosidase alfa in LOPD patients. The LOTS clinical trial confirmed the stabilization of motor and pulmonary functions, which also correlated with reduced lysosomal glycogen levels after ERT in the EMBASSY study^7^. (Table 1). Despite being a breakthrough therapy of its time, a recent 10-year follow-up study found that a mild to moderate improvement in LOPD patients may be observed during the first few years, followed later by a plateau, or decline in patient outcomes, raising significant concerns regarding its long-term use^8^. It is widely believed that limited biodistribution of rhGAA and low expression of the CI-M6P receptors on skeletal muscle cells, causing decreased intracellular uptake, are responsible for reduced long-term therapeutic efficacy of alglucosidase alfa in LOPD patients^9^. (Fig. 2).

**Table 1:** Current enzyme replacement therapies for Pompe disease

Study ID	Drug	Phase	Sample size	Outcomes	Adverse effect
(COMET) TRIAL (NCT02782741)^11^	100 Patients were randomly allocated avalglucosidase alfa (*n*=51) or alglucosidase alfa (*n*=49).	Phase 3	100 patients at 55 sites in 20 countries (aged ≥3 years) with enzymatically confirmed late-onset Pompe disease who had never received treatment^11^.	The primary and secondary outcomes were change from baseline to week 52 in 6-min walk distance (6MWD) and sitting forced vital capacity (FVC). Treatment with avalglucosidase alfa resulted in a least-squares mean improvement in upright FVC% predicted of 2·89% compared with 0·46% with alglucosidase alfa at week 49^11^.	In the avalglucosidase alfa group, 23 (45%) of 51 participants reported treatment-emergent adverse events, while in the alglucosidase alfa group, 24 (49%) of 49 participants reported. Thirteen (26%) and sixteen (33%) of the avalglucosidase alfa and alglucosidase alfa groups, respectively, developed infusion-associated reactions. Four of the five trial withdrawals—two of which were related to infusion—were caused by adverse events, all of which occurred in the alglucosidase alfa group^11^.
(LOTS) TRIAL (NCT00158600)^7^	Patients who qualified were randomly assigned in a ratio of 2:1 to receive biweekly infusions of alglucosidase alfa (20 mg per kilogram of body weight) or placebo for 78 weeks^7^.	Phase 3	Ninety patients (assigned to either alglucosidase alfa (60 patients) and placebo (30 patients). Of this group, 81 completed the study) who were 8 years of age or older, ambulatory, and free of invasive ventilation were randomly assigned at eight centers in the United States and Europe^7^.	The primary outcomes were distance walked during a 6-minute walk test and percentage of predicted forced vital capacity (FVC). Secondary and tertiary efficacy end points included changes in the percentage of the predicted QMT leg score and QMT arm score, maximum inspiratory pressure, and maximum expiratory pressure. By 78 weeks, treatment with alglucosidase alfa had significantly increased both the distance walked on the 6-minute walk test and the percentage of the predicted FVC with a mean increase of 25.1 m on the 6-minute walk test whereas the placebo group had a decrease of 3.0 m, for an estimated differential treatment effect of 28.1 m (*P*=0.03)^7^.	Majority of adverse events had mild to moderate severity with similarities in the reported events (headache, nasopharyngitis, and falls) between the groups. 28% of patients receiving alglucosidase alfa and 23% of those receiving a placebo experienced infusion-associated event. Five to eight percent of patients receiving alglucosidase alfa experienced anaphylactic, allergic, and infusion-associated events, which included flushing, hyperhidrosis, urticaria, chest pain, vomiting, and elevated blood pressure^7^.

van der Ploeg AT, *et al*. 2010^7^.

Diaz-Manera J, *et al*. 2021^18^.

### Avalglucosidase

To enhance the limited therapeutic efficacy of alglucosidase, a solution was developed by designing avalglucosidase, a novel rhGAA, produced by a chemical process where an oligosaccharide carrying bis-M6P residues was conjugated to recombinant human GAA through oxime chemistry, resulting in enhanced affinity for the CI-M6P receptors and broader distribution in systemic tissues^10^. A recent randomized, double-blind clinical trial (COMET) evaluated its therapeutic efficacy and safety by comparing it to glucosidase. Following favorable results from the COMET trial, avalglucosidase gained approval in 2021 to treat LOPD, with a recommended dose of 20 mg/kg body weight, administered once every two weeks^11^. (Table 1) (Fig. 2).

## POMBILITI and OPFOLDA: First two-component therapy for LOPD

**Figure 3:**
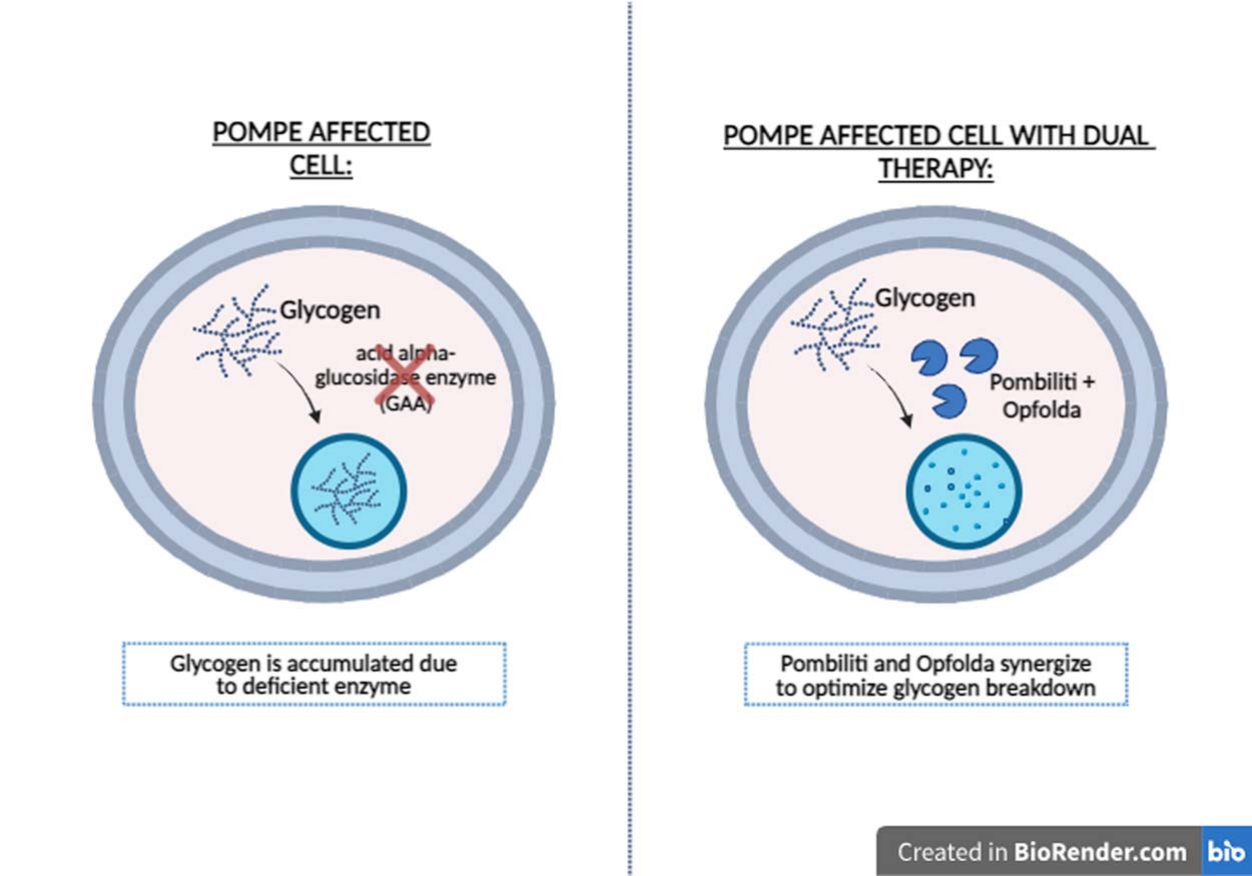
Pombiliti + Opfolda in Pompe disease. (Created with biorender.com).

POMBILITI + OPFOLDA, a dual-component therapy, represents a significant breakthrough in treating late-onset Pompe disease for adults who have not experienced improvements from existing treatments. This therapy pairs POMBILITI, a rhGAA enriched with bis-M6P that improves cellular uptake while preserving enzymatic function, with OPFOLDA. POMBILITI possesses the same amino acid sequence as the natural GAA enzyme but features complex-type N-glycan structures with two M6P moieties on the same glycan. These M6P moieties facilitate binding to M6P receptors on the cell surface. OPFOLDA, coadministered with POMBILITI, is a synthetic analog of D-glucose and the active ingredient in Zavesca, approved for treating adult patients with mild to moderate Type 1 Gaucher disease when ERT is not viable. OPFOLDA binds to, stabilizes, and prevents the inactivation of POMBILITI in the bloodstream after infusion^12^. As a result, this oral enzyme stabilizer minimizes enzyme activity loss in the bloodstream. After cellular uptake, POMBILITI is transferred to lysosomes, where it replaces the deficient or defective lysosomal GAA enzyme and breaks down accumulated glycogen, thereby preventing the sequelae associated with lysosomal glycogen buildup, accounting for the clinical presentation of Pompe disease. Conversely, OPFOLDA does not undergo cellular uptake and remains in the bloodstream^1,13^. By preventing the breakdown of POMBILITI before its uptake by cells, it effectively maintains serum levels of POMBILITI with a relatively much lower dose than previously available treatment options^14^. (Fig. 3).

## Key discoveries from the PROPEL trial

The FDA’s approval for Pombiliti in conjunction with Opfolda is based on findings from Trial 1/NCT03729362, involving 123 late-onset Pompe disease (LOPD) patients aged 18 and above across 61 sites in 24 countries, including the U.S. Patients were randomly assigned to receive Opfolda with Pombiliti or a placebo alongside a non-U.S.-approved alglucosidase alfa product biweekly for 52 weeks. Most patients had prior alglucosidase alfa treatment (ERT-experienced), while some were treatment-naïve. Those receiving Opfolda and Pombiliti demonstrated more favorable changes in sitting forced vital capacity (FVC) and 6-min walk distance (6MWD) compared to the placebo group. Specifically, they showed increased sitting FVC and improved 6MWD at Week 52. ERT-experienced patients also experienced positive changes in these parameters with Opfolda and Pombiliti. This research underscores the potential benefits of this treatment combination for eligible LOPD patients, representing a significant advancement in their treatment options^15–18^. (Table 2).

**Table 2:** Key discoveries from the PROPEL Trial

Study ID	Drug	Phase	Sample size	Outcomes	Adverse effect
(PROPEL) Trial (NCT03729362)^17^.	Patients were randomized 2:1 to receive POMBILITI in combination with Opfolda or a non-U.S.-approved alglucosidase alfa product with placebo every other week for 52 weeks^17^.	Phase 3	123 late-onset Pompe disease (LOPD) patients aged 18 and above across 61 sites in 24 countries	The primary and secondary outcomes were change from baseline to week 52 in 6-min walk distance (6MWD) and sitting forced vital capacity (FVC)^17^.	The most common adverse reactions (≥5%) were headache and diarrhea and (2%) of patients reported myalgia, arthralgia, increased blood pressure, pain, tremor, dyspepsia, asthenia, constipation, infusion site swelling, flank pain, malaise, paresthesia, and decreased platelet count^17^.

Schoser B, *et al*. 2021^17^.

## Recommended dosage and side effects

OPFOLDA (cipaglucosidase alfa-atga) and POMBILITI (miglustat) are vital components of a treatment plan. It is imperative to administer OPFOLDA alongside POMBILITI, commencing this combined therapy two weeks after the last ERT dose. For OPFOLDA, a 65 mg capsule should be taken ~1 h before the intravenous infusion of POMBILITI. It is crucial to refrain from consuming other food or drinks for at least two hours before and after taking OPFOLDA. The POMBILITI infusion should be initiated about an hour after OPFOLDA ingestion. The recommended dosage for POMBILITI is 20 mg per kilogram of body weight, given via a 4-h intravenous infusion every other week. Maintaining this precise treatment schedule is essential for its effectiveness^18^.

An analysis was conducted on the safety of POMBILITI and OPFOLDA treatment in 151 adult patients with LOPD across three clinical trials. These trials included 85 participants in a randomized, double-blind, active-controlled trial (Trial 1), 37 individuals in an open-label extension study that transitioned from a non-U.S.-approved alglucosidase alfa product to POMBILITI with OPFOLDA, and 29 subjects in an open-label trial. Trial 1 evaluated the benefits and side effects of POMBILITI in combination with Opfolda, and all three trials assessed the side effects of POMBILITI in combination with Opfolda^16,18^.

The analysis unveiled those severe adverse reactions, including anaphylaxis and urticaria, observed in two or more patients who received POMBILITI with OPFOLDA. Furthermore, five patients discontinued POMBILITI treatment due to adverse reactions, with four ceasing the therapy due to severe adverse reactions. The most prevalent adverse reactions (occurring in at least 5% of patients) within this combined patient group were headache, diarrhea, fatigue, nausea, abdominal pain, and pyrexia^16,18^.

**Figure 4:**
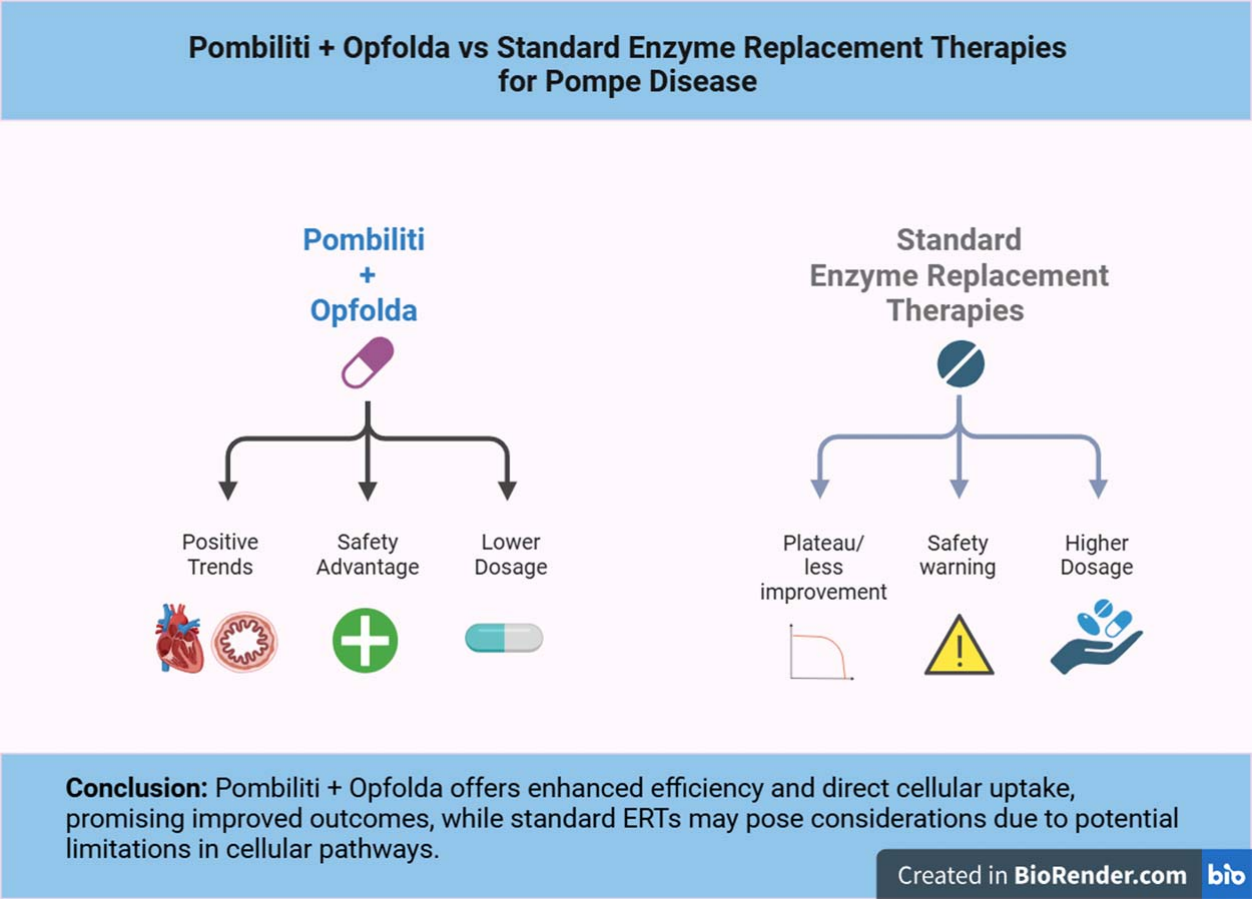
Pombiliti + Opfolda vs. standard enzyme replacement therapies for Pompe disease. (Created with biorender.com).

Significantly, 32% of patients in these trials experienced infusion-associated reactions (IARs) during their treatment with POMBILITI combined with OPFOLDA. These reported IARs encompassed a variety of symptoms, including headache, myalgia, diarrhea, nausea, fatigue, muscle spasms, pyrexia, dizziness, cough, chills, rash, vomiting, dyspnea, pain, abdominal distension, tachycardia, urticaria, flatulence, pruritus, abdominal pain, chest discomfort, flushing, hyperhidrosis, dysgeusia, and hypertension. These findings offer valuable insights into the safety profile of POMBILITI in conjunction with OPFOLDA for individuals with LOPD^16,18^. Fig. 4 compares the effectiveness and safety of Pombiliti + Opfolda versus Standard Enzyme Replacement Therapies (ERTs) for Pompe Disease. It highlights that Pombiliti + Opfolda offers positive trends, a safety advantage, and a lower dosage, whereas standard ERTs show plateaued improvement, safety warnings, and require higher dosages.

## Future applications and limitations

Late-onset Pompe disease, an uncommon yet severe lysosomal disorder, poses significant challenges with its characteristic symptoms, including skeletal muscle weakness and progressive respiratory complications. Despite strides in medical research, the Pompe community continues to grapple with limited treatment choices, leaving unmet needs prevalent. The recent FDA approval of Pombiliti and Opfolda underscores the remarkable synergy of scientific innovation and our unwavering dedication to enhancing the lives of Pompe disease patients. For those affected by late-onset Pompe disease, the approval of Pombiliti and Opfolda signifies a long-awaited turning point, offering hope and advocacy for expanded therapeutic options. Clinical investigations have revealed promising advancements in musculoskeletal strength and respiratory function with the combination administration of Pombiliti and Opfolda. This breakthrough opens new doors for treatment and propels us towards a future where the challenges posed by Pompe disease can be more effectively addressed^19^.

Nevertheless, it’s imperative to recognize the comprehensive safety profile of this treatment, which includes a Boxed Warning underscoring potential risks such as embryo-fetal toxicity, infertility, lactation, hypersensitivity reactions (including anaphylaxis), and infusion-associated reactions (IARs). It’s important to note that the combination of OPFOLDA and POMBILITI is not recommended during pregnancy. Given these safety considerations, healthcare professionals and patients should exercise caution and diligence when employing this therapy to address late-onset Pompe disease (LOPD)^16,18^.

To enhance the treatment of late-onset Pompe disease (LOPD) with Pombiliti and Opfolda, several strategies can be implemented. For optimal dosing, Pombiliti should be administered intravenously at 20 mg/kg every other week, about an hour after taking Opfolda orally, with specific timing adjustments if a dose is missed. Combining this regimen with antihistamines, antipyretics, and corticosteroids can help reduce hypersensitivity reactions. Clinical trials have shown that this combination improves patient mobility, potentially lowering long-term healthcare costs and enhancing quality of life. Ensuring drug accessibility through ongoing research, clinical trials, and monitoring of adverse reactions in diverse populations is crucial. Additionally, tracking antidrug and neutralizing antibodies will help assess the long-term safety and efficacy of the treatment. These measures aim to maximize the benefits and accessibility of Pombiliti and Opfolda for LOPD patients.

## Conclusion

In summary, enzyme replacement therapies are the primary method for treating Pompe disease. However, studies have shown that over a long period, patients’ conditions often stabilize and then deteriorate, necessitating progressively higher doses of standard ERTs or the consideration of new therapeutic options. The approval of the first two-component therapy, comprising POMBILITI plus OPFOLDA, brings fresh hope for individuals with LOPD who have reached a plateau or show a decline in health condition with existing treatment. Clinical studies have demonstrated noteworthy enhancements in musculoskeletal and respiratory parameters when utilizing POMBILITI and OPFOLDA. Nevertheless, further research is required to assess its long-term outcomes, primarily to determine whether patient outcomes still improve after the initial few years of beginning the treatment. While the approval of this first dual-component therapy marks a significant milestone in Pompe disease management, ongoing research is essential to develop more effective combinations with improved therapeutic efficacy and tolerance to enhance patients’ quality of life with Pomp disease. Furthermore, clinical trials like the PROPEL trial should be conducted to assess the short-term and long-term effects of this therapy on IOPD patients, as they face even more significant management challenges.

## Ethical approval

No patient was involved in this type of study, therefore no ethical approval was required.

## Consent

The study is an editorial and was not done on patients or volunteers, therefore no written consent was required.

## Source of funding

Not applicable.

## Author contribution

Conceptualization and project administration: A.S. and R.R. Original draft of manuscript: A.S., R.R., L.S., L. Reviewing and editing the manuscript: A.A. Visualization: R.R.

## Conflicts of interest disclosure

The authors declare no conflicts of interest.

## Research registration unique identifying number (UIN)

1. Name of the registry: Not applicable.
2. Unique Identifying number or registration ID: Not applicable.
3. Hyperlink to your specific registration (must be publicly accessible and will be checked): Not applicable.

## Guarantor

Rumaisa Riaz, Aymar.

## Data availability statement

Not applicable.

## Provenance and peer review

Not commissioned, externally peer-reviewed.
